# Fusiform face area development correlates with development in higher-order social brain regions

**DOI:** 10.1016/j.dcn.2026.101765

**Published:** 2026-06-18

**Authors:** Lorena Jiménez-Sánchez, Melissa Thye, Hilary Richardson

**Affiliations:** Department of Psychology, School of Philosophy, Psychology and Language Sciences, University of Edinburgh, Edinburgh, UK

**Keywords:** Face selectivity, development, social cognition, fusiform face area, paediatric fMRI

## Abstract

The fusiform face area (FFA) preferentially responds to faces within the first months of life. One hypothesis is that higher-order social responses in middle medial prefrontal cortex (MMPFC) or face responses in superior temporal sulcus (STS) drive the development of face-selective responses in FFA, with right-hemisphere dominance in FFA eventually arising from lateralised connections to these regions. Another hypothesis proposes an innate face template in the amygdala guides attention to face-like shapes. This study opportunistically examined the development of the FFA, MMPFC, STS, and amygdala in childhood using an open cross-sectional movie-viewing fMRI dataset with 3–12-year-olds (N = 117, M = 6.77 years) and adults (N = 33, M = 24.77 years). We tested for correlations between FFA development and development in MMPFC, STS, and amygdala on the premise that associations between these regions may be observable even in children, and such associations could constrain hypotheses and analytic approaches in future studies with infants. First, we measured functional maturity- i.e., how similar each child’s response to the movie was to an adult average response timecourse. In all regions, older children’s responses were more adult-like. Next, we tested whether FFA maturity correlated with functional connectivity with, or functional maturity of, MMPFC, STS, or amygdala. Children with more mature right FFA responses showed stronger right FFA-right MMPFC connectivity. Children with more mature FFA responses also had more mature STS responses, bilaterally. This study provides preliminary evidence that FFA co-develops with higher-order social brain regions.

## Highlights

• What drives face-selective responses in FFA is the subject of recent debate.
• 117 children aged 3–12 years watched a short movie while undergoing fMRI.
• Right FFA development correlated with functional connectivity to right MMPFC
• FFA development correlated with STS development, bilaterally.
• FFA codevelops with higher-order social brain regions (controlling for age).

## Graphical Abstract

**Figure:**
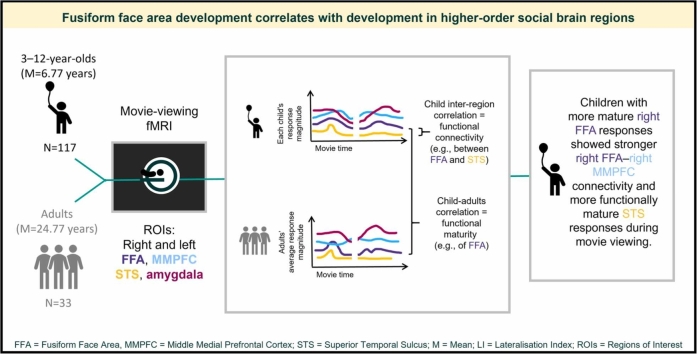

## 1 Introduction

Faces are salient stimuli in our environment and an important source for recognising and learning about other people. In adults, a network of cortical regions respond preferentially to faces (Haxby et al., 2000, Kanwisher et al., 1997), including bilateral superior temporal sulcus (STS), occipital face area, and fusiform face area (FFA); these responses are more dominant in the right hemisphere (Behrmann and Plaut, 2020, Kanwisher et al., 1997). In particular, the right FFA demonstrates the most robust face-selective activation relative to control stimuli (e.g., objects, or scenes) in adults (Downing and Chan, 2006, Grill-Spector et al., 1998, Kanwisher et al., 1997) and functional connectivity between face regions is stronger in the right hemisphere from infancy (Lesinger et al., 2023). The FFA appears to have face-selective responses by two months of age (Kosakowski et al., 2024), with no observable effect of age among 2–9-month-old infants. However, there is also evidence that FFA face selectivity continues to increase during childhood, primarily through reduced responses to other visual categories (Cantlon and Pinel, 2011, Gomez et al., 2017, Natu et al., 2016, Nordt et al., 2021, Peelen et al., 2009). Here, we conduct opportunistic analyses in a functional magnetic resonance imaging (fMRI) dataset of 3–12-year-old children to investigate neural correlates of FFA development and provide insight into plausible drivers of face-selective FFA responses.

Hypotheses about the developmental drivers of face-selective responses in FFA have recently focused on explaining the emergence of face-selective responses in infants. Powell et al. (2018) articulated three non-mutually exclusive hypotheses which draw from empirical evidence across the lifespan. The first hypothesis proposes that infants choose to look at faces to engage in positive, self-relevant social interactions. In this account, responses to positive, self-relevant social stimuli in middle medial prefrontal cortex (MMPFC) drive attention to faces and consequently the development of face-selective FFA responses. MMPFC responds to socially relevant stimuli (e.g., familiar faces or voices, mutual gaze) in adults (Amodio and Frith, 2006, Gobbini and Haxby, 2007, Van Den Bos et al., 2007) and infants (Grossmann et al., 2008, Imafuku et al., 2014, Naoi et al., 2012, Urakawa et al., 2015), and shows face-selective responses in two-month-old infants (Kosakowski et al., 2024). The MMPFC may be involved in face processing from birth: an electroencephalography (EEG) study in newborns reported face-specific neural responses that were localised to several cortical regions, including MMPFC (Buiatti et al., 2019). There is also evidence for “top-down” influences from MMPFC and STS to FFA responses in adults asked to switch between reporting the emotional valence and the age of a face (Anzellotti et al., 2017). This hypothesis could plausibly explain right-hemisphere dominance for face processing, which could arise from laterally-biased connectivity with “higher-order” regions including MMPFC (Powell et al., 2018), similar to how pre-existing connectivity to higher-order language regions explains the left-lateralised development of the visual word form area (Op De Beeck et al., 2019, Saygin et al., 2016). More recent theoretical accounts propose that hemispheric asymmetries emerge through an interaction between such neural constraints and experience viewing faces (Behrmann et al., 2025). Infant fMRI studies have yet to detect right-lateralised face responses (Kosakowski et al., 2024), but a right-hemisphere bias in face responses has been observed in school-aged children and adults (Kanwisher et al., 1997, Liu et al., 2024; also see Thome et al., 2022).

The second hypothesis described by Powell et al. (2018) is that the brain contains an innate, subcortical “face template” that directs infants’ attention to face-like shapes (Morton and Johnson, 1991), driving the development of face-selective cortical regions. While there is currently no direct evidence for a subcortical “face template”, studies with adults support the existence of a rapid subcortical route for face processing involving the amygdala (Krolak-Salmon et al., 2004). This route mainly reflects subcortical responses to low spatial frequencies of visual stimuli (Vuilleumier et al., 2003) which may influence cortical processing, including in FFA (Morris et al., 1998). However, many of the aforementioned results involve experiments using fearful faces, so it remains unclear if amygdala responses reflect matches between face stimuli and a face template, or processing of other aspects of stimuli, such as fear (Johnson, 2005), emotional valence (Liu et al., 2022), salience and/or biological/social relevance (Adolphs, 2008). Very little research investigates amygdala responses in human infants, likely because it is not possible to measure subcortical responses using functional near-infrared spectroscopy (fNIRS) or EEG - which are more tolerant to participant motion than fMRI. To our knowledge, only one study has used fMRI to measure amygdala responses to faces in infancy. While fMRI affords the high spatial resolution and full-brain coverage necessary for studying the amygdala, results were inconclusive (Kosakowski et al., 2021). Kosakowski et al. reported difficulties parcellating the amygdala using structural MRI data, given poor tissue contrast in infants, and difficulties coregistering the parcellated amygdala with awake infant fMRI data (given time-resolution trade-offs made during the development of awake infant fMRI sequences). Because subcortical responses are smaller in magnitude than cortical responses, the consequences of these methodological challenges are exacerbated. It remains possible that the amygdala responds to faces even in infants, but these responses have not yet been detected. In children, the amygdala shows functional connectivity with the right FFA by six years of age (Feng et al., 2022) and bilateral amygdala and FFA respond to faces independent of facial expressions in adults (Kale et al., 2019).

The third hypothesis described by Powell et al. (2018) suggests that infants' visual experiences co-activate distinct neural populations with preferences for different low-level visual image statistics (e.g., rectilinearity vs curvilinearity), and repeated co-activation over time drives the development of preferential responses to faces (Arcaro et al., 2017, Livingstone et al., 2017). Parents often interact with their infants face-to-face, so infants acquire extensive experience with face images on their fovea at a close distance. Neurons in the extrastriate cortex with preferences for foveal input, curvilinearity, and low spatial frequency are then repeatedly co-activated over time and, perhaps, become selective for face stimuli in virtue of these characteristics. This effect has been demonstrated in childhood, where retinal eccentricity and repeated experience with certain visual stimuli result in shared functional topography across individuals (Gomez et al., 2019). However, this mechanism predicts bilaterally symmetric face responses, so this hypothesis cannot yet explain (eventual) right-lateralised face responses.

While evidence for early emerging face-selective responses in infants is compelling (Deen et al., 2017, Kosakowski et al., 2024), there is also strong evidence for continued development in face-selective responses in FFA in childhood (Cantlon and Pinel, 2011, Cohen et al., 2019, Golarai et al., 2007, Gomez et al., 2017, Scherf et al., 2007). The hypotheses reviewed above, primarily proposed in the context of emerging face-selective responses in infants, make distinct neural predictions that could plausibly also support continued FFA development in children. For example, if right FFA specialisation is driven by e.g., connectivity to “top-down” regions such as MMPFC, this causal history might be observable as concurrent associations between FFA development and its functional connectivity to MMPFC in children. This premise is supported by machine learning studies showing that resting-state connectivity can successfully predict task-based brain activity in children (Bernstein-Eliav and Tavor, 2024) and by concurrent associations between network-level functional maturation and connectivity in other social brain regions among children (Richardson, 2019).

We conducted opportunistic analyses of naturalistic movie-viewing data to test predictions of the first two hypotheses and investigate neural correlates of face-selective responses in the right FFA of 3–12-year-old children. In preregistered analyses, we characterised functional responses of bilateral FFA, MMPFC, and amygdala to faces and tested whether functional maturity of FFA correlates with its functional connectivity to MMPFC and amygdala. Our prediction was based on prior empirical evidence from other “social brain” networks. Specifically, prior work shows that functional connectivity within theory-of-mind regions increases with age, and that children with more strongly connected networks exhibit more functionally mature i.e., “adult-like” responses during movie viewing (Richardson, 2019, Richardson et al., 2018). Although no prior work directly linked functional connectivity or maturity to functional selectivity in a single region, all measures appear to increase across development (Bowman et al., 2015, Richardson, 2019, Richardson et al., 2018). Primary analyses focused on correlates of right FFA development, given evidence for right-hemisphere dominance for face-selective responses (Behrmann and Plaut, 2020, Kanwisher et al., 1997). Non-preregistered analyses tested for correlations between STS and FFA development; the STS is another higher-order face-selective region that could plausibly shape development of FFA (Anzellotti et al., 2017, Deen et al., 2017, Kosakowski et al., 2024). In additional non-preregistered analyses, we examined correlations between functional maturity of the right FFA and bilateral MMPFC, amygdala, and STS.

We acknowledge two limitations of our approach upfront: first, cross-sectional evidence cannot reveal causal mechanisms of development. Correlational evidence can motivate future research testing for causal relationships and so is often a useful and practical starting point. Second, evidence in support of any of the hypotheses articulated above in children does not imply that the same developmental mechanisms are responsible for the initial emergence of face-selective responses in infants; different developmental mechanisms could be at work at different ages. Nonetheless, understanding neural correlates of continued FFA development is of interest to the field (Cantlon and Pinel, 2011, Cohen et al., 2019, Golarai et al., 2007, Gomez et al., 2017, Scherf et al., 2007) and could plausibly motivate further research across the lifespan. We return to these limitations and their implications in the Discussion.

## 2 Materials and methods

This study involved opportunistic analyses of an open fMRI dataset downloaded from OpenNeuro (https://openneuro.org/datasets/ds000228; Richardson et al., 2018). Analyses were preregistered on the Open Science Framework (https://osf.io/hytsr). Deviations are reported where relevant and summarised in Table S1.

### 2.1 Participants and data

The dataset includes children (N = 122; ages 3.5–12 years) and adults (N = 33; ages 18–39 years). Five children were excluded from analyses for preprocessing issues (segmentation error likely due to a priori defacing issues, n = 1) or not meeting participant motion exclusion criteria (see section 2.4.2, n = 4). The final sample included 117 children (M(SD) = 6.77(2.33) years of age; 62 females) and 33 adults (M(SD) = 24.77(5.31) years of age; 20 females, Table 1).

**Table 1:** Demographic characteristics of participants by age group.

**Age Group**	**N**	**Age Range** **M (SD)**	**Gender** **(F/M)**	**Handedness** **(R/L/Ambi)**	**Raw IQ** **M (SD)**	**Scaled/standard IQ** **M (SD)**
**3yo**	15	3.53–3.99 3.78 (0.17)	10/5	13/2/0	15.71 (3.47)	10.50 (2.68)
**4yo**	14	4.06–4.86 4.43 (0.29)	8/6	13/0/1	16.93 (3.91)	9.64 (3.39)
**5yo**	32	5.01–5.99 5.51 (0.29)	15/17	26/5/1	20.41 (5.23)	112.03 (13.58)
**7yo**	23	7.00–7.96 7.54 (0.37)	11/12	23/0/0	29.43 (6.80)	116.74 (16.78)
**8–12yo**	33	8.02–12.30 9.80 (1.18)	18/15	32/1/0	35.39 (3.62)	119.18 (10.71)

Nonverbal IQ was measured via the WPPSI block design task for children ages 3–4 years and through the KBIT−2 matrices task for children ages 5–12 years - for more information, see original publication (Richardson et al., 2018). F/M = Female/Male; M = Mean; R/L/Ambi = Right/Left/Ambidextrous; SD = Standard Deviation; yo = year old.

All participants were recruited from the local community surrounding Massachusetts Institute of Technology (MIT) (Cambridge, MA, USA), gave written consent (parent/guardian consent and child assent was received for all child participants), and had normal or corrected-to-normal vision. Recruitment and experiment protocols were approved by the Committee on the Use of Humans as Experimental participants (COUHES) at MIT.

Primary analyses were conducted using data from children; data from adults were used to identify timepoints for response magnitudes to face and scene events and to calculate functional maturity in children (sections 2.4.5 and 2.4.6).

### 2.2 fMRI stimuli

All participants watched a silent version of “Partly Cloudy” (Reher and Sohn, 2009), a 5.6-min animated movie. A short description of the plot can be found online (https://www.pixar.com/partlycloudy#partly-cloudy−1). The stimulus was preceded by 10 s of rest and subtended 17.62 × 13.07° of visual angle.

### 2.3 fMRI data acquisition

Data acquisition procedures and parameters have been described previously (Richardson et al., 2018). Briefly, children completed a mock scan to acclimate to the scanner and learn to stay still. Young participants could hold a stuffed animal for comfort and to reduce movement. During scanning, an experimenter stood near the bore to ensure children stayed awake, attended to the movie, and remained still.

Whole-brain structural and functional MRI data were acquired using a 3-Tesla Siemens Tim Trio scanner at the Athinoula A. Martinos Imaging Centre at MIT. Children under age 5 used one of two custom 32-channel phased-array head coils made for younger (N = 3, M = 3.91, SD = 0.42 years old in the public dataset) or older children (N = 28, M = 4.07, SD = 0.42 years old in the public dataset). Older participants used the standard Siemens 32-channel head coil (Richardson et al., 2018). The only difference between head coils was their size. These size-optimised head coils have been shown to increase signal-to-noise in participants with smaller heads (Keil et al., 2011). T1-weighted structural images were collected with 1 mm isotropic voxels. Functional data were obtained in a single run with a gradient-echo EPI sequence sensitive to Blood Oxygen Level Dependent (BOLD) contrast in 32 interleaved near-axial slices aligned with the anterior/posterior commissure, and covering the whole brain (repetition time, TR: 2 s; 168 volumes). Participants were recruited for different studies and had small differences in voxel size and slice gaps, so all functional data were up-sampled in normalised space to 2 mm isotropic voxels. Prospective acquisition correction was used to adjust for head motion one TR back (Thesen et al., 2000).

### 2.4 fMRI data analysis

#### 2.4.1 Preprocessing

Data were preprocessed using fMRIPrep 24.0.0 (Esteban et al., 2019, Markiewicz et al., 2024) which is based on Nypipe 1.8.6 (Gorgolewski et al., 2011). Briefly, data were run through the complete fMRIPrep workflow, which includes brain-skull separation, brain tissue segmentation, spatial normalisation, and confound estimation (see Appendix S1 for detailed description). Data were then smoothed using a Gaussian filter with a 5 mm kernel.

#### 2.4.2 Motion treatment

FMRIPrep outputs were visually inspected and subjected to a quality control workflow to exclude data with excessive participant movement prior to first-level modelling. Outlier volumes were defined as those with (1) framewise displacement (FD) > 1 and (2) using the following thresholds within rapidart, the nipype implementation of the ART toolbox: more than 1 mm composite motion relative to the previous volume or a fluctuation in global signal exceeding 3 standard deviations from the mean global signal. If one-third or more of the volumes within the functional run were identified as motion outliers, participants were excluded from analyses (n = 4 children). Although preregistered, the threshold of standardised DVARS > 1.5 was not applied, as it was not an analysis-informed threshold and resulted in the exclusion of participants with otherwise acceptable data quality (https://osf.io/hytsr); these participants have been included in prior publications using this dataset (Richardson et al., 2018, Yates et al., 2021). Mean FD and mean DVARS were uncorrelated with age among children (Spearman correlation tests: r_s_(115) = −0.06 and r_s_(115) = 0.11, respectively; Fig. S1). Mean FD was included as a covariate in statistical analyses to account for participant motion (see section 2.5).

#### 2.4.3 Region of interest (ROI) definition

We analysed responses in subject-specific left and right FFA, MMPFC, amygdala and STS ROIs (8 ROIs total). Analyses of STS were conducted after our preregistered analyses with other ROIs were completed; we did not preregister analyses of STS. FFA, MMPFC and STS ROIs were defined functionally using a procedure described previously (Kamps et al., 2022). In this approach, the average response timecourse from localiser-defined functional ROIs (fROIs) in independent adult samples (“reference timecourses”) are regressed on movie timecourses for each child. This provides an estimate of how similar a voxel’s timecourse is to the adult reference timecourse. Subject-specific fROIs are then defined as the highest-ranking voxels to contrasts of previously extracted, localiser-defined fROI timecourses chosen to reflect the domain-selectivity of each region. Relative to approaches that use particular movie scenes as events (e.g., face events, scene events) to define contrasts, this approach leverages the full response timecourse for ROI definition. Here, left and right FFA ROIs (defined with faces > objects in the original localiser task with adults) were defined as the top 80 voxels to the contrast of (left or right) FFA > (left or right) lateral occipital cortex (LOC) timecourses, within large left and right FFA search spaces (Julian et al., 2012). Left and right MMPFC ROIs (defined with false beliefs > false photographs in the original localiser task with adults) were defined as the top 80 voxels to the contrast of (left or right) MMPFC > (left or right) secondary somatosensory cortex (S2), within a large bilateral MMPFC search space (Dufour et al., 2013; https://saxelab.mit.edu/use-our-theory-mind-group-maps/) split by hemisphere (for left and right MMPFC search spaces, see https://osf.io/mxkag/). We targeted middle MPFC (MMPFC) rather than dorsal or ventral MPFC based on reviewing whole-brain maps of responses to faces in individual infants reported previously (e.g., Fig. 3 in Kosakowski et al., 2024). Left and right STS ROIs (defined with faces > objects in the original localiser task with adults) were defined as the top 80 voxels to the contrast of (left or right) STS > (left or right) LOC timecourses, within large left and right STS search spaces (Julian et al., 2012). We chose 80 voxels based on prior studies examining cortical responses to facial, emotional, and social features (Kliemann et al., 2018, Richardson et al., 2020, Richardson et al., 2023, Skerry and Saxe, 2014). Further details about the adult reference timecourses used for fROI definition are provided in Appendix S2**.** First-level modelling carried out for fROI definition included rapidart-identified outlier volumes, the top five aCompCor components and adult reference timecourses for all localiser-defined fROIs as additional regressors; all timecourses were included in one model, as previously (Kamps et al., 2022). Data were high-pass filtered (0.01 Hz).

Because the open dataset contains only one run of movie-viewing per participant, we used a split-half fROI definition approach (Kamps et al., 2022), in which the movie was divided into first and second halves to allow independent fROI definition and response extraction. Subject-specific fROIs were defined in one movie half (using one half of the reference timecourses) and response timecourses used for analyses were extracted in the opposite half; this procedure was iterated such that response timecourses from both movie halves were analysed.

Unlike the MMPFC, STS, and FFA, the amygdala is a distinct anatomical structure. Given this, left and right amygdala ROIs were defined anatomically within individual participants using automatic segmentation by FreeSurfer Aseg Atlas (Fischl et al., 2002) within fMRIPrep. We targeted the amygdala to understand the influence of a rapid subcortical route for face processing in FFA development. Although this route includes other regions such as the superior colliculus (Johnson et al., 2015, Mcfadyen et al., 2019, Morton and Johnson, 1991), we focused on the amygdala because it shows functional connectivity with the right FFA in children by six years of age (Feng et al., 2022) and its larger size and clearer anatomical boundaries make it more tractable for human neuroimaging. Further research is needed to replicate and extend these findings to other subcortical structures.

#### 2.4.4 Timecourse extraction

Preprocessed, smoothed timecourses were high-pass filtered (0.01 Hz), outlier volumes were interpolated with cubic spline interpolation and censored, and the top five aCompCor were regressed out. Timecourses were then averaged across voxels within an ROI and z-scored, resulting in one timecourse per subject-specific ROI.

For fROIs (left and right FFA, MMPFC and STS), timecourses for each movie half were extracted from the fROI defined in the other, independent half of the movie. The middle 8 s of the timecourse were excluded to prevent temporal autocorrelation across movie halves; note that the number of seconds excluded was increased from the preregistered value (6 s; https://osf.io/hytsr) to obtain two movie halves of equal duration (given TR = 2 s).

For anatomically-defined left and right amygdala ROIs, full movie response timecourses were extracted and analysed.

#### 2.4.5 Functional maturity

Functional maturity was defined as the z-scored Pearson correlation between each child’s timecourse and the average adult timecourse per ROI. For FFA, MMPFC and STS, the reference average adult timecourses were obtained from localiser-defined fROIs (FFA and STS: n = 13 adults, available at https://osf.io/7a8w5/; MMPFC: n = 17 adults, available at https://osf.io/mxkag/). Functional maturity in these regions was calculated separately for the first and second halves of the movie, and the two values were averaged to produce a single measure per ROI. For amygdala, the reference average adult timecourses were based on anatomically defined ROIs (n = 33 adults), and functional maturity was computed using the entire movie. We used adult timecourses as the reference for computing functional maturity based on prior developmental neuroimaging work showing that the similarity between children’s and adults’ neural responses is related to cognitive development and the functional specialisation of (social) brain regions (Cantlon and Pinel, 2011, Richardson, 2019, Richardson et al., 2018).

#### 2.4.6 Response magnitude to faces and scenes

Since functional maturity does not capture information about responses to faces, we also measured the magnitude of response to face events in our ROIs. Scene events were used as a negative control (dispreferred stimulus). We extracted z-scored response magnitude values from the peak timepoint of scenes that evoked reliable, positive responses from FFA (face events) or parahippocampal place area (PPA, scene events) in adults - as defined in prior research using the same adult sample studied here (n = 33; Kamps et al., 2022). There were 12 face events/timepoints and 12 scene events/timepoints. The peak timepoint was defined as the single timepoint (1 TR = 2 s) that evoked the highest average response in adults.

#### 2.4.7 Inter-region correlations (i.e., functional connectivity)

For each child participant, inter-region correlations (IRCs) were defined as the z-scored Pearson correlations between each ROI timecourse. For IRCs involving fROIs (left and right FFA, left and right MMPFC, and left and right STS), IRCs were calculated per movie half and averaged to obtain one measure per ROI. For IRCs only involving amygdala ROIs, IRCs were calculated using the full movie response timecourse.

### 2.5 Statistical analyses

Data analyses were conducted and plots generated using R, version 4.4.3 (R Core Team, 2023). See Table S2 for statistical model formulae.

#### 2.5.1 *Developmental change in* functional responses of FFA, MMPFC, amygdala and STS

We tested for an effect of age on the functional maturity of each ROI using linear mixed-effects models with age, hemisphere, motion (mean FD) and an age-by-hemisphere interaction as fixed effects, alongside a random intercept for participant.

We also tested if the magnitude of response to face events in each ROI correlated with age using linear mixed-effects models. Peak magnitude for events per ROI were used as the outcome; one regression was conducted per event. Kamps et al. (2022) identified 12 face events and 12 scene events (F01-F12 and S01-S12, respectively, following their numbering convention). We tested for fixed effects of age, hemisphere, motion and an age-by-hemisphere interaction effect, alongside a random intercept for participant. We conducted a regression per event, rather than testing for effects on the average response magnitude across events per event type, because some events may be more sensitive to age-related differences than others and averaging across events could obscure age effects rather than increase power. We did not use mixed-effects models including responses to all events for similar reasons and because we did not expect to be sensitive to event-by-age interaction effects. Given the number of events and tests, results were corrected for multiple comparisons using a Bonferroni approach: effects were considered significant at α = 0.004 for face and scene events (p = 0.05/12, for 12 events per ROI).

We also investigated if functional connectivity between ROIs associated with age using a combination of linear models for within-region connectivity and linear mixed-effects models for between-region connectivity. Functional connectivity was the outcome in all models. For within-region connectivity (e.g., between left and right FFA ROIs), we tested for fixed effects of age and motion. For between-region connectivity (e.g., between right FFA and left/right MMPFC ROIs or right FFA and left/right amygdala ROIs), we tested for fixed effects of age, hemisphere, motion and an age-by-hemisphere interaction, alongside a random intercept for participant. Three primary models included connectivity of right FFA as the dependent variable; six secondary models included connectivity of left FFA, left and right MMPFC and amygdala as the dependent variable.

After conducting preregistered analyses, we conducted the same analyses for STS: we tested for age effects on functional maturity of STS, whether responses in STS to face and scene events associated with age, and whether functional connectivity between right FFA (primary models) and other ROIs (secondary models) and left and right STS increased with age.

#### 2.5.2 Associations between functional maturity of FFA and its functional connectivity to MMPFC, amygdala and STS

A key hypothesis we aimed to test was whether functional maturity of FFA correlated with its functional connectivity to MMPFC or amygdala (https://osf.io/hytsr). Our preregistered primary analysis used linear mixed-effects models with functional maturity of right FFA as dependent variable, functional connectivity between right FFA and the MMPFC, between right FFA and amygdala, participant’s age, hemisphere (left or right for MMPFC and amygdala), motion and an age-by-hemisphere-by-ROI interaction (and all embedded two-way interactions) as fixed effects, alongside a random intercept for participant. As a secondary analysis, we preregistered a similar model with functional maturity of left FFA as dependent variable. However, these models did not adequately converge and may have led to unstable estimates.

Instead, two separate, non-preregistered linear mixed-effects models were conducted to examine the association between right FFA functional maturity and each functional connectivity measure of interest. In each regression, functional connectivity between (1) right FFA and right or left MMPFC, (2) right FFA and right or left amygdala was the dependent variable with fixed effects of functional maturity of right FFA, age, motion and an age-by-hemisphere-by-functional maturity interaction (and all embedded two-way interactions), and a random intercept for participant. All non-significant interaction terms were removed from the final regression. Secondary models tested the same relationships for functional connectivity of left FFA to other ROIs and functional maturity of left FFA. In these models, the main dependent and independent variables were reversed relative to the preregistered plan because it is not possible to run a regression with repeated measures within a predictor (i.e., functional connectivity, which included a value per hemisphere, could not be an independent variable). Because all tests and claims are correlational, this reversal does not impact our conclusions. Given the hypotheses we set out to test, we also conducted a non-preregistered analysis testing for a hemisphere-specific association - i.e., between right FFA functional maturity and its functional connectivity specifically to right MMPFC. We used a linear model with functional maturity of right FFA as dependent variable and fixed effects of functional connectivity between right FFA and right MMPFC, age, motion and an age-by-hemisphere-by-functional connectivity interaction (and all embedded two-way interactions).

We also ran non-preregistered linear mixed-effects models testing the same kind of associations, in the same way, using connectivity between right FFA and STS as dependent variable. The STS contains an early-developing face-selective region that could also plausibly shape or codevelop with FFA (Anzellotti et al., 2017, Deen et al., 2017, Kosakowski et al., 2024). Secondary models tested the same relationships for functional connectivity between left FFA and STS and functional maturity of left FFA instead of right FFA.

In additional non-preregistered analyses, we examined associations between the functional maturity of the right FFA, MMPFC, amygdala, and STS. We repeated the three linear mixed-effect models used previously, this time including the functional maturity of the MMPFC, amygdala, and STS as dependent variables. Secondary models tested the same relationships with functional maturity of left FFA as the independent variable.

#### 2.5.3 Preregistered analyses of lateralisation of FFA face-response and its correlates

We preregistered analyses to test associations between lateralisation of the FFA face-response and functional connectivity with or maturity of MMPFC and amygdala (https://osf.io/hytsr). Our lateralisation index was defined using the relative response to face and scene events, per hemisphere. However, because faces appeared throughout nearly all movie scenes - including scene events - these events did not provide a clear estimate of face-selective responses. Thus, we were not confident in our lateralisation index and include detailed methods and results in supplementary materials (Appendix S3).

## 3 Results

### 3.1 Development of FFA face response and functional connectivity

**Fig. 1:**
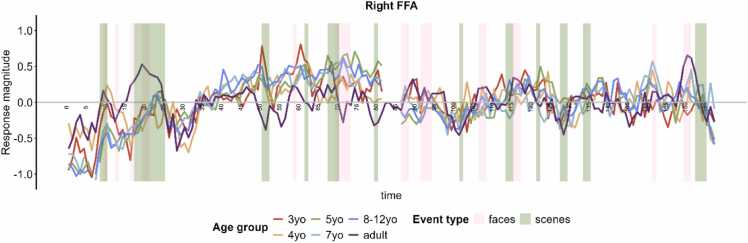
Average timecourse of right FFA during viewing of "Partly Cloudy" by age group (3yo: n = 15, 4yo: n = 14, 5yo: n = 32, 7yo: n = 23, 8–12yo: n = 33, adults: n = 33). Each timepoint along the x-axis corresponds to a single TR (2 s). Shaded blocks show timepoints identified as face (pink) and scene (green) events in a reverse correlation analysis conducted on adults in prior research (Kamps et al., 2022). N = 117 children, N = 33 adults. See Fig. S2 for timecourses of all bilateral regions. FFA = Fusiform Face Area; yo = year old.

**Fig. 2:**
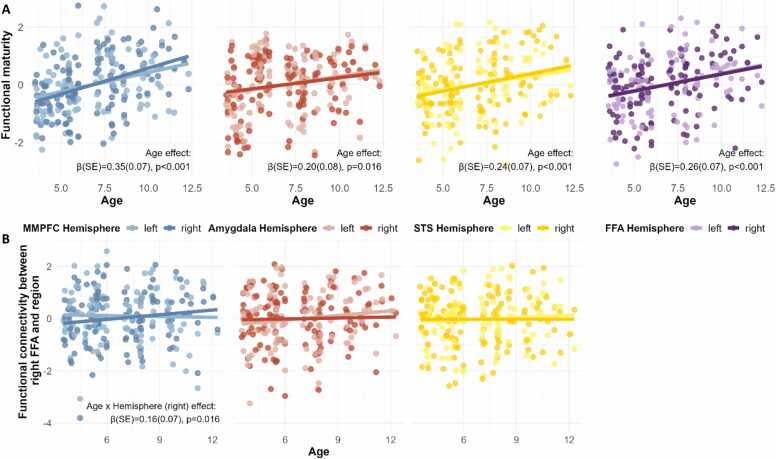
A) Association between age (x axis) and functional maturity (y axis) of MMPFC, amygdala, STS and FFA. B) Association between age (x axis) and functional connectivity between right FFA and MMPFC, right FFA and amygdala, or right FFA and STS (y axis). Blue indicates relationships with MMPFC, red indicates relationships with amygdala, yellow indicates relationships with STS. Lighter colours correspond to the left hemisphere, darker colours correspond to the right hemisphere. In all scatterplots, lines represent linear regression fits estimated using the least-squares method; significant main effects are noted. N = 117. FFA = Fusiform Face Area; MMPFC = Middle Medial Prefrontal Cortex, STS = Superior Temporal Sulcus; SE = Standard Error.

We first investigated developmental change in functional maturity of FFA, MMPFC, amygdala and STS. Older children had more functionally mature (i.e., adult-like) FFA responses, controlling for motion (age effect: β(SE) = 0.26(0.07), p = 1.836 × 10^−4^, Table S3, Fig. 1, Fig. 2A, S2A). Similar age effects on functional maturity were observed in MMPFC (β(SE) = 0.35(0.07), p = 4.841 × 10^−6^, Table S3, Fig. 2A), amygdala (β(SE) = 0.20(0.08), p = 0.016, Table S3, Fig. 2), and STS (β(SE) = 0.24(0.07), p = 3.968 × 10^−4^, Table S3, Fig. 2).

Since the functional maturity measure does not capture information about face responses, we tested if the magnitude of response to face events changed with age. FFA response magnitude did not vary with age for any face or scene event (defined in adults), controlling for motion and correcting for multiple comparisons (α=0.004 for face and scene events, tested separately; Fig. 1, S2A). However, MMPFC response magnitude decreased with age for one face event (F05; β(SE) = −0.23(0.06), p = 3.798 × 10⁻⁴; see asterisk in Fig. S2B, and Fig. S3 for interpretation), depicting Gus removing porcupine spikes from Peck’s head (evoking physical pain). In contrast, STS response magnitude increased with age for another face event (F10; β(SE) = 0.14(0.04), p = 7.311 × 10⁻⁴; see asterisk in Fig. S2D, and Fig. S3), showing Gus scolding the crocodile. No other regions showed significant age effects for face or scene events. Full results for all events and ROIs are provided in Table S4.

We also investigated if functional connectivity between ROIs changed with age. Functional connectivity between right FFA and other ROIs were generally positive across all children (right FFA and right MMPFC: M(SD) Pearson’s correlation value = 0.19(0.23), t = 8.73, p = 2.193 × 10⁻^1^⁴; right FFA and left MMPFC: M(SD) Pearson’s correlation value = 0.11(0.24), t = 4.91, p = 3.049 × 10^⁻6^; right FFA and right amygdala: M(SD) Pearson’s correlation value = 0.34(0.19), t = 19.05, p = 1.654 × 10⁻^37^; right FFA and left amygdala: M(SD) Pearson’s correlation value = 0.23(0.21), t = 11.89, p = 8.178 × 10⁻^22^, right FFA and right STS: M(SD) Pearson’s correlation value = 0.38(0.20), t = 20.41, p = 3.353 × 10⁻^40^; right FFA and left STS: M(SD) Pearson’s correlation value = 0.17(0.20), t = 9.03, p = 4.463 × 10⁻^15^; all *t*-tests against zero, non-preregistered, Fig. S4). While functional connectivity between the right FFA and bilateral MMPFC did not increase with age (β(SE) = −0.01(0.09), p = 0.873, Table S5, Fig. 2B, S4), there was a significant age-by-hemisphere interaction such that connectivity between right FFA and right MMPFC increased more with age than connectivity between right FFA and left MMPFC (β(SE) = 0.16(0.07), p = 0.016, Table S5, Fig. 2B, S4). In secondary analyses, connectivity between the right MMPFC and bilateral STS increased with age (β(SE) = 0.15(0.07), p = 0.038, Table S5, Fig. 2B, S4). There were no other age effects or interactions on functional connectivity between left and right FFA, MMPFC, amygdala, and STS (all ps ≥ 0.127, Table S5, Fig. 2B, S4, S5).

### 3.2 Functional maturity of FFA: Associations with functional connectivity with or functional maturity of MMPFC, amygdala, or STS

**Fig. 3:**
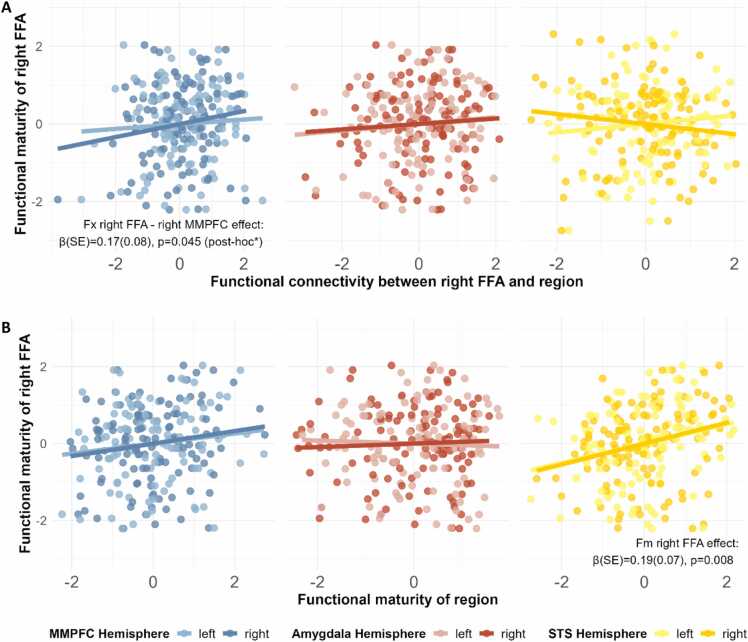
A) Association between functional connectivity (x axis) of right FFA and MMPFC (blue), amygdala (red), or STS (yellow) and functional maturity of right FFA (y axis). *Post-hoc model statistics reported for this panel. B) Association between functional maturity (x axis) of MMPFC (blue), amygdala (red), and STS (yellow) and functional maturity of right FFA (y axis). Blue indicates relationships with MMPFC, red indicates relationships with amygdala, yellow indicates relationships with STS. Lighter colours correspond to the left hemisphere, darker colours correspond to the right hemisphere of MMPFC, amygdala, and STS. In all scatterplots, lines represent linear regression fits estimated using the least-squares method; significant main effects are noted. N = 117. FFA = Fusiform Face Area; MMPFC = Middle Medial Prefrontal Cortex; STS = Superior Temporal Sulcus; Fm = Functional Maturity; Fx = Functional Connectivity; SE = Standard Error.

A key question we aimed to address was whether FFA functional maturity was associated with its functional connectivity to the MMPFC or the amygdala. We did not observe an association between more functionally mature (i.e., "adult-like") right FFA responses and stronger functional connectivity between the right FFA and bilateral MMPFC or amygdala (all ps ≥ 0.060, Table 2**A**, Fig. 3**A**), or (in non-preregistered analyses) bilateral STS (p = 0.106, Table 2**A**, Fig. 3**A).** Because connectivity between right FFA and right MMPFC increased more with age than connectivity between right FFA and left MMPFC and including left MMPFC could obscure right-hemisphere-specific associations between the functional maturity of the right FFA and its connectivity to MMPFC, we conducted post-hoc analyses specifically testing for an association within the right hemisphere. In this analysis, children with more functionally mature right FFA responses had stronger functional connectivity between the right FFA and right MMPFC (β(SE) = 0.17(0.08), p = 0.045, Table 2**B**). To explore if participant motion moderated the association between functional connectivity of right FFA and right MMPFC, we tested for an interaction effect between mean FD and functional connectivity between the right FFA and right MMPFC in the previous model (see Table S2 for non-preregistered model specifications). There was no evidence that participant motion moderated the association between functional maturity of the right FFA and functional connectivity between the right FFA and right MMPFC (interaction term: β(SE) = −0.09(0.09), p = 0.281).

**Table 2:** Associations between functional maturity of right FFA and functional connectivity between FFA, MMPFC, amygdala, and STS.

***A***						
***Fx right FFA - MMPFC***						
Predictors	β		SE	z	p	
(Intercept)	0.11		0.09	1.24	0.216	
Fm right FFA	0.18		0.09	1.90	0.060	
MMPFC hemisphere (right)	−0.09		0.07	−1.35	0.179	
Age	−0.06		0.09	−0.62	0.536	
Mean FD	0.20		0.09	2.15	**0.034**	
Age x Hemisphere (right)	0.16		0.07	2.44	**0.016**	
Random effects	σ					
ID	0.84					
Residual	0.51					

***Fx right FFA - amygdala***						
Predictors	β		SE	z	p	
(Intercept)	−0.01		0.10	−0.07	0.941	
Fm right FFA	0.09		0.10	0.91	0.366	
Amygdala hemisphere (right)	0.01		0.08	0.11	0.909	
Age	0.06		0.09	0.70	0.487	
Mean FD	0.06		0.09	0.63	0.532	
Random effects	σ					
ID (σ)	0.84					
Residual (σ)	0.63					

***Fx right FFA - STS***						
Predictors	β		SE	z	p	
(Intercept)	−0.01		0.09	−0.14	0.890	
Fm right FFA	0.12		0.08	1.63	0.106	
STS hemisphere (right)	−0.01		0.12	−0.08	0.940	
Age	0.02		0.07	0.23	0.817	
Mean FD	0.19		0.07	2.65	**0.009**	
Random effects	σ					
ID (σ)	0.36					
Residual (σ)	0.90					

***B***						
***Fm right FFA***						
Predictors	β	SE		z		p
(Intercept)	0.00	0.08		−0.05		0.963
Fx right FFA - right MMPFC	0.17	0.08		2.03		**0.045**
Age	0.22	0.08		2.61		**0.010**
Mean FD	−0.36	0.08		−4.28		**3.863 × 10**^**−5**^

A) Age-by-hemisphere-by-functional maturity interaction terms (and embedded two-way interaction terms) for each test were not statistically significant and were excluded from the final models. B) The interaction term of age-by-functional maturity for the test was not statistically significant and excluded from the final model. Reported βs are standardised, ps < 0.05 are shown in bold. N = 117. FFA = Fusiform Face Area; MMPFC = Middle Medial Prefrontal Cortex; STS = Superior Temporal Sulcus; Fm = Functional Maturity; Fx = Functional Connectivity; FD = Framewise Displacement; SE = Standard Error.

In secondary analyses of left FFA, children with more functionally mature left FFA responses had weaker functional connectivity between the left FFA and bilateral STS (β(SE) = −0.14(0.07), p = 0.046, Table S6, Fig. S6A).

Finally, we sought to understand if these regions matured in tandem. In non-preregistered analyses, we did not observe evidence that children with more functionally mature right FFA responses had more functionally mature bilateral MMPFC or amygdala responses (all ps ≥ 0.544, Table 3, Fig. 3**B**), controlling for age. However, functional maturity of right FFA associated with functional maturity of bilateral STS, controlling for age (β(SE) = 0.19(0.07), p = 0.008, Table 3, Fig. 3**B**). There was no evidence that participant motion moderated the association between functional maturity of the right FFA and STS (interaction term: β(SE) = −0.01(0.07), p = 0.840, see Table S2 for non-preregistered model specifications). The same pattern was observed for the left FFA (Table S7, Fig. S6B) - suggesting that our analyses were sensitive to associations between functional maturity of different regions.

**Table 3:** Associations between functional maturity of right FFA and functional maturity of MMPFC, amygdala, and STS.

***Fm MMPFC***				
Predictors	β	SE	z	p
(Intercept)	0.00	0.09	0.00	1.000
Fm right FFA	−0.02	0.08	−0.19	0.851
MMPFC hemisphere (right)	0.00	0.09	0.00	1.000
Age	0.35	0.08	4.66	**8.615 × 10**^**−6**^
Mean FD	−0.18	0.08	−2.28	**0.025**
Random effects	σ			
ID	0.63			
Residual	0.68			

***Fm amygdala***				
Predictors	β	SE	z	p
(Intercept)	0.00	0.09	0.00	1.000
Fm right FFA	−0.06	0.09	−0.61	0.544
Amygdala hemisphere (right)	0.00	0.08	0.00	1.000
Age	0.21	0.08	2.51	**0.014**
Mean FD	0.00	0.09	0.01	0.995
Random effects	σ			
ID (σ)	0.76			
Residual (σ)	0.64			

***Fm STS***				
Predictors	β	SE	z	p
(Intercept)	0.00	0.09	0.00	1.000
Fm right FFA	0.19	0.07	2.72	**0.008**
STS hemisphere (right)	0.00	0.12	0.00	1.000
Age	0.19	0.07	2.91	**0.004**
Mean FD	−0.04	0.07	−0.64	0.522
Random effects	σ			
ID (σ)	0.20			
Residual (σ)	0.93			

Age-by-hemisphere-by-functional maturity interaction terms (and embedded two-way interaction terms) for each test were not statistically significant and were excluded from the final models. Reported βs are standardised, ps < 0.05 are shown in bold. N = 117. FFA = Fusiform Face Area; MMPFC = Middle Medial Prefrontal Cortex; STS = Superior Temporal Sulcus; Fm = Functional Maturity; FD = Framewise Displacement; SE = Standard Error.

Correlations among functional connectivity measures and among functional maturity measures are presented in Figs. S7 and S8, respectively.

## 4 Discussion

Evidence from fMRI studies with awake infants (Deen et al., 2017, Kosakowski et al., 2024) has fostered debate concerning drivers of face-selective responses in the right FFA (Livingstone et al., 2017, Op De Beeck et al., 2019, Powell et al., 2018). Here, we conducted opportunistic analyses on an existing cross-sectional paediatric fMRI dataset to test predictions of two non-mutually exclusive hypotheses articulated by Powell et al. (2018): that face-selective responses in right FFA develop alongside (1) higher-order brain systems involved in social interaction - in particular, the MMPFC; and/or (2) subcortical regions which plausibly contain a face template - i.e., the amygdala. To test these hypotheses, we examined whether the functional connectivity between FFA and MMPFC or amygdala correlated with the functional maturity of right FFA. Children with stronger functional connectivity between right FFA and right MMPFC had more functionally mature right FFA responses during movie-viewing. We extended our approach to study associations between FFA and STS; and observed that children with more functionally mature STS responses had more functionally mature FFA responses, bilaterally. All analyses controlled for effects of age. Together, this evidence suggests that FFA co-develops with higher-order social brain regions, including MMPFC and STS.

To test for an association between higher-order social brain regions and FFA development, our preregistered analyses focused on MMPFC, given its early involvement in processing self-relevant social interactions (Grossmann, 2025, Saxe and Kosakowski, 2025) and evidence for face-selective responses in MMPFC in infants (Deen et al., 2017, Kosakowski et al., 2024). The association we observed between functional maturity of the right FFA and functional connectivity between right FFA and right MMPFC was modest in magnitude compared with prior findings linking functional maturity of the theory-of-mind network with within-network connectivity (Richardson, 2019). Associations between functional maturity and connectivity may be easier to detect at the network level because timecourses of individual fROIs are noisy relative to network averages. Interestingly, we did not observe evidence for a direct association between the functional maturity of right FFA and right MMPFC - despite being sensitive to this kind of association between right FFA and bilateral STS. We extended our analyses to include STS because both MMPFC and STS exert “top-down” influences on FFA responses in adults (Anzellotti et al., 2017). The STS contains an early-developing face-selective subregion (Deen et al., 2017, Kosakowski et al., 2024), which is particularly responsive to dynamic faces in adults (Pitcher et al., 2011). The face-selective subregion of STS appears to be distinct from the subregion involved in social interaction perception (Deen and Koldewyn, 2015, Im et al., 2025, Masson and Isik, 2021) and has been suggested to be important for facial expression recognition (Sliwinska and Pitcher, 2018). In children, prolonged development in face-selective STS (Kamps et al., 2022, Tansey et al., 2023), entails increased functional correlations with higher-order “theory-of-mind” regions, including MMPFC (Kamps et al., 2022). Our results suggest that this prolonged development also correlates with development in FFA. We also observed that children with more “adult-like” *left* FFA responses had *weaker* functional connectivity between left FFA and STS; this could reflect the development of non-face-related responses in left FFA (i.e., visual word form area development) with age (Behrmann et al., 2025, Centanni et al., 2018). Our interpretations of the relationship between FFA and STS development remain speculative and require direct testing in future studies.

Our positive evidence for an association between right FFA development and its functional connectivity with right MMPFC (and functional maturity of STS) does not diminish the potential role of the amygdala in FFA development. Null results are difficult to interpret; while our approach was sensitive to associations between functional maturity and connectivity of other ROIs, effects involving the amygdala (if present) could be smaller/harder to detect. Interestingly, functional connectivity was numerically stronger between right FFA and amygdala, relative to FFA and MMPFC (and strongest between right FFA and STS). It is possible that the associations we observed between FFA development and its connectivity to MMPFC and STS development reflect a prolonged developmental trajectory of MMPFC and STS and our sensitivity to developmental variability in these measures within the age range of our sample. Future research could investigate if the amygdala contributes to the emergence of face-selective responses in infants and explore the role of other subcortical structures, like the superior colliculus (Johnson et al., 2015, Morton and Johnson, 1991).

Additionally, our results do not provide evidence against the hypothesis that face-selective FFA responses develop through repeated co-activation of neurons tuned to low-level visual features of faces (Arcaro et al., 2017, Livingstone et al., 2017, Powell et al., 2018). One challenge to this hypothesis raised by prior research is that this account, on its own, would predict bilateral face-selective FFA responses - and so it is difficult to resolve with known right-lateralised face responses in FFA (Powell et al., 2018). However, it remains unclear if early face-selective FFA responses are right-lateralised (Kosakowski et al., 2024), highlighting the possibility that neural mechanisms that drive the right-lateralisation of face responses may be different from those that support the initial development of face-selective responses in FFA. Our experimental design did not enable us to confidently measure the lateralisation of face-selective responses (Appendix S3; though note that, consistent with Lesinger et al., 2023, functional connectivity between FFA and MMPFC, amygdala, and STS was numerically larger - i.e., stronger - between right hemisphere regions than left hemisphere regions). Future studies should investigate the developmental trajectory of FFA face-response lateralisation and its relationship with face selectivity. More broadly, it is likely that multiple factors shape the development of face-selective FFA responses and all three hypotheses are worth interrogating in future research.

Our results are limited in several ways. First, opportunistic analyses of existing datasets are, by definition, not optimised for testing hypotheses other than those that the researchers had in mind when initially acquiring the data - and so should often be considered an initial step in a line of research. Second, given that our analyses were conducted with a cross-sectional dataset of 3–12-year-old children, associations could reflect directional relationships, whereby MMPFC/STS play a causal role in the development of face responses in FFA, or where FFA is a driver of face selectivity in MMPFC, supporting its development as a social region. Our results could also simply reflect co-development of functional connectivity and maturity of these regions (beyond common effects of age) - or even common effects of development in a different brain region, not included in the present analyses. Further, associations between these different metrics could reflect a historical relationship: i.e., these measures would explain the initial development of face-selective FFA responses early in life, or they could simply reflect concurrent development among older children. More studies are needed to tease apart these alternatives, but such research will be challenging. For example, a micro-genetic study could measure these aspects of brain development concurrently as face-selective responses emerge (Dehaene-Lambertz et al., 2018) - but this would likely require multiple fMRI sessions per infant within the first months of life (Kosakowski et al., 2024, O’doherty et al., 2026). Causal relationships between these different aspects of brain development could be informed through controlled rearing studies with non-human primate infants (Livingstone et al., 2017) or intervention studies investigating effects of positive, meaningful social interactions on attention to faces (Chawarska et al., 2025, Hayward et al., 2018) and FFA development. It may also be important for future research to employ interactive designs (Piazza et al., 2020) to measure brain responses related to self-relevant, socially meaningful interactions. Extending our approach into adolescence, a period of robust face selectivity but also substantial developmental change of brain networks including the FFA, may reveal neural mechanisms underlying individual differences in face selectivity (Golarai et al., 2010). Finally, it is worth noting that defining fROIs in children as the subset of voxels with the most “adult-like” functional response to the movie may have excluded voxels with face-selective responses that differed in some way from adult responses, potentially underestimating developmental change in face responses. We chose to define fROIs in this way given the similar spatial organisation of face-selective responses across the lifespan (Kosakowski et al., 2024) and the benefits of using subject-specific functional responses to define fROIs (Kamps et al., 2022, Saxe et al., 2006) our analyses were sensitive to developmental effects despite this choice. Nonetheless, future research might consider ways to capture neural correlates of differences or development of face processing among children (Gomez et al., 2017) in addition to capturing resemblance to mature responses. The results of our opportunistic study help to constrain hypotheses and motivate experimental design and analytic choices for this future “planned” research.

In conclusion, face-selective responses in FFA appear to be correlated with development of higher-order brain regions including MMPFC and STS in 3–12-year-old children. Future studies using longitudinal and intervention designs, socially relevant, self-referential stimuli, and experimental designs that allow for measuring the lateralisation of face-selective responses earlier in development are needed to clarify the mechanisms underlying the emergence and right-lateralisation of face-selective responses in the FFA.

## Funding

For the purposes of open access, the author has applied a Creative Commons Attribution (CC BY) licence to any Author Accepted Manuscript version arising from this submission.

LJ-S was supported by the University of Edinburgh Wellcome Trust Translational Neuroscience 4-year PhD programme (Grant No. 108890/Z/15/Z). The funding sources had no role in the study design, execution, analysis, interpretation of the data, decision to publish or preparation of the manuscript.

## CRediT authorship contribution statement

**Lorena Jiménez-Sánchez:** Writing – review & editing, Writing – original draft, Visualization, Methodology, Investigation, Funding acquisition, Formal analysis, Data curation, Conceptualization. **Melissa Thye:** Writing – review & editing, Software, Methodology, Formal analysis, Data curation. **Hilary Richardson:** Writing – review & editing, Supervision, Software, Resources, Project administration, Methodology, Investigation, Data curation, Conceptualization.

## Declaration of Generative AI and AI-assisted technologies in the writing process

During the preparation of this work, the author(s) used ChatGPT to assist with cleaning scripts written in R as part of the data analysis process. After using this tool, the author(s) reviewed and edited the output as necessary and take full responsibility for the content of the published article.

## Declaration of Competing Interest

The authors declare that they have no known competing financial interests or personal relationships that could have appeared to influence the work reported in this paper

## Data Availability

The movie fMRI data analysed during the current study (originally collected by Richardson et al., 2018) are publicly available on OpenNeuro (https://openneuro.org/datasets/ds000228). Further details about the fMRI analysis pipeline, and publicly available code, can be found at https://github.com/hrichardsonlab/fmri-analysis. Average timecourses from the referent adult populations, used as regressors in this study, are available through OSF (https://osf.io/7a8w5/ for FFA and LOC, and https://osf.io/mxkag/ for MMPFC and S2). Search spaces are also available through OSF (https://osf.io/mxkag/ for MMPFC and S2 split by hemisphere, and left FFA defined by flipping the right FFA search space). Data and scripts used for this analysis can be accessed at https://github.com/LorenaJS/FRIC.
